# Men's Suicidal thoughts and behaviors and conformity to masculine norms: A person-centered, latent profile approach

**DOI:** 10.1016/j.heliyon.2024.e39094

**Published:** 2024-10-09

**Authors:** Lukas Eggenberger, Lena Spangenberg, Matthew C. Genuchi, Andreas Walther

**Affiliations:** a Experimental Pharmacopsychology and Psychological Addiction Research, Department of Adult Psychiatry and Psychotherapy, University Hospital of Psychiatry and University of Zurich, Zurich, Switzerland; b Jacobs Center for Productive Youth Development, University of Zurich, Zurich, Switzerland; c Department of Medical Psychology and Medical Sociology, University Leipzig, Leipzig, Germany; d Department of Psychological Science, Boise State University, Boise, ID, USA; e Clinical Psychology and Psychotherapy, Psychological Institute, University of Zurich, Zurich, Switzerland

**Keywords:** Men, Masculinities, Traditional masculinity ideologies, Depression, Suicide, Suicidal thoughts and behaviors

## Abstract

**Background:**

Men are up to four times more likely to die by suicide than women. At the same time, men are less likely to disclose suicidal ideation and transition more rapidly from ideation to attempt. Recently, socialized gender norms and particularly conformity to masculine norms (CMN) have been discussed as driving factors for men's increased risk for suicidal thoughts and behaviors (STBs). This study aims to examine the individual interplay between CMN dimensions and their association with depression symptoms, help-seeking, and STBs.

**Methods:**

Using data from an anonymous online survey of 488 cisgender men, latent profile analysis was performed to identify CMN subgroups. Multigroup comparisons and hierarchical regression analyses were used to estimate differences in sociodemographic characteristics, depression symptoms, psychotherapy use, and STBs.

**Results:**

Three latent CMN subgroups were identified: Egalitarians (58.6 %; characterized by overall low CMN), Players (16.0 %; characterized by patriarchal beliefs, endorsement of sexual promiscuity, and heterosexual self-presentation), and Stoics (25.4 %; characterized by restrictive emotionality, self-reliance, and engagement in risky behavior). Stoics showed a 2.32 times higher risk for a lifetime suicide attempt, younger age, stronger somatization of depression symptoms, and stronger unbearability beliefs.

**Conclusion:**

The interplay between the CMN dimensions restrictive emotionality, self-reliance, and willingness to engage in risky behavior, paired with suicidal beliefs about the unbearability of emotional pain, may create a suicidogenic psychosocial system. Acknowledging this high-risk subgroup of men conforming to restrictive masculine norms may aid the development of tailored intervention programs, ultimately mitigating the risk for a suicide attempt.

## Highlights

• Conformity to masculine gender ideologies is associated with increased suicide risk
• Emotional restrictivity and risky behavior raise men's suicide risk 2.32-fold
• Some men perceive their emotional pain as more unbearable than others do
• Men somatizing depression symptoms creates a challenge for health practitioners
• Considering masculinity ideologies may enhance tailored suicide prevention

## 1 Introduction

Men are typically found to be between two to four times more likely to die by suicide than women [1,2]. At the same time, men are less likely to disclose suicidal thoughts to health care professionals [3,4] and they progress more rapidly from suicidal thoughts to suicidal behavior [5]. However, the processes underlying suicidal thoughts and behaviors (STBs) are thought to be complex and not fully understood. For example, suicidal thoughts do not necessarily progress in a linear fashion to suicidal behaviors [[6], [7], [8]], they are highly time-sensitive with large fluctuations over short periods of time [[9], [10], [11]], and they may not always precede suicidal behavior [12,13]. Consequently, frequently discussed risk factors for STBs such as depressive disorders, suicidal ideation, or prior suicide attempts are nonspecific and provide little to no predictive value for future suicide attempts [[14], [15], [16]]. In contrast, risk factors arising from the need to conform to social norms and beliefs about masculinity may provide an alternative perspective on men's increased suicide risk [17]. For instance, beliefs on masculinity which were conceptualized prior to second-wave feminism (i.e., traditional masculinity ideologies; TMI) often emphasize the importance of men being self-reliant, in control of their emotions, and not displaying any vulnerabilities [[18], [19], [20], [21]]. Behaviors consistent with TMI (i.e., conformity to masculine norms; CMN) have been linked to potentially suicidogenic behaviors such as maladaptive externalizing depression symptoms [[22], [23], [24]] and a reluctance to seek help when experiencing psychological distress [[25], [26], [27], [28]]. Recent studies have also highlighted more direct associations between STBs and constructs closely related to TMI, such as status loss, self-reliance, and stoicism [[29], [30], [31], [32], [33]]. However, the role of conformity to these dimensions within the broader psychosocial context of men warrants further quantitative investigation [[34], [35], [36], [37]]. Thus, the present study will try to disentangle the complex relationship between CMN and men's increased risk for suicide employing a person-centric methodological approach.

### 1.1 Frequently discussed risk factors

Because men are affected disproportionately by suicide death, a need exists to better understand the causes of suicidal behavior among men and advance effective preventative measures. Mental health disorders, particularly depressive disorders, constitute an important category of risk factors for STBs. It has been estimated that about nine out of ten people who die by suicide have been diagnosed with a psychiatric disorder at time of their suicide [38,39]. Among those diagnoses, affective or mood disorders, such as depression, have been found to show the strongest association with STBs [15,40]. Among men, depressive disorders may be an underestimated risk factor, as there seems to exist a subgroup of young men who die by suicide but were never diagnosed with a mental health disorder and, similarly, never visited a mental health professional prior to their suicide death [34,41].

Another consistently and unanimously discussed risk factor for a suicide attempt is suicidal ideation [14,40,42]. However, suicidal ideation is a very heterogenous construct. For example, while most people are in control over their suicidal thoughts, others tend to not disclose any suicidal ideation or even imminent suicide plans [43]. In a case-control study by Smith et al. [44], 85 % of depressive patients who died of suicide have denied prior suicidal ideation. Similarly, Wastler et al. [12] reported that about 54 % of US adults with a recent suicide attempt denied prior active suicidal thoughts, and about 23 % denied any prior suicidal thoughts. Notably, men seem to be particularly unlikely to disclose suicidal ideation to health care professionals [3,4]. Lastly, suicidal ideation is often very unstable over time, with large fluctuations sometimes occurring during the course of a single day or even within a few hours [9,10].

Due to the lack of stability and reliability of suicidal ideation, increasingly holistic approaches have been proposed by looking at more stable constructs such as suicidal or suicidogenic beliefs, rather than ideation. Suicidogenic beliefs can be understood as a person's perception of or belief about themselves that can lead to suicidal ideation [7]. Prominent suicidogenic beliefs involve, for example, being a burden to others or feeling socially disconnected [45], feelings of hopelessness and unbearable distress [46], or feeling defeated or entrapped [47]. Rudd [48] further proposed to combine the three suicidogenic belief categories unlovability, unsolvability, and unbearability into one single, overreaching suicidogenic belief system. This belief system is thought to be even more persistent than fleeting affective-cognitive states (e.g., being a burden or feeling defeated) and may thus provide a more reliable and stable assessment of not only suicidogenic beliefs but also suicide risk in general [[49], [50], [51]].

Even though mental health disorders as well as suicidal ideation and beliefs have frequently been studied and discussed as driving factors for increased suicide risk, they seem to offer almost no explanatory and predictive value for future suicidal behavior [[14], [15], [16]]. Because suicidal behavior is considered to be highly dependent on the individual's context [52,53], a paradigm shift towards understanding the complex and diverse pathways leading to suicidal ideation and behavior is warranted [8,54]. Specifically, recent literature highlights the importance of considering and linking individual components and processes associated with suicide risk to construct formal suicide theories [[54], [55], [56]]. Such holistic approaches may be used to better understand the psychosocial framework of men's increased risk for suicidal behavior [17,57]. In particular, societal and individual beliefs about masculinity provide an important context in which men's risk for suicidal behavior is elevated [36,57].

### 1.2 CMN as a novel risk factor

Masculine gender role norms, so-called traditional masculinity ideologies (TMI), can be understood as thoughts or beliefs about how a typical man should be and behave [18,19,58,59]. These beliefs are culturally and socially defined constructs that, during their conceptualization prior to the early 1960s, served to assert men's dominant position in a hegemonic and patriarchal society [20,21]. Thus, typical TMI portray men as physically strong, in control, and in contrast to behaviors traditionally viewed as feminine, for example, expressing feelings of vulnerability or showing signs of affection [18,19,59,60]. Conformity to masculine norms (CMN) is thought to be partially internalized through socialization processes at an early age, where non-conformity is typically more harshly sanctioned in boys than in girls [61,62].

Conformity to TMI that portray men as stoic, strong, and invulnerable stands in stark contrast with experiencing depressive symptoms such as sadness or reduced self-esteem [27,63]. Consequently, some men tend to avoid or mask depressive symptoms by exhibiting externalizing symptoms, such as anger, substance abuse, or somatic symptoms, which have been linked to increased risk for suicidal behavior and suicide death [[64], [65], [66], [67]]. Furthermore, men's presentation of atypical, externalizing depressive symptoms can lead to them not being recognized by conventional diagnostic instruments [23,68,69]. In turn, this subgroup of potentially depressed men may not receive the support that they need, which puts them at even greater risk for externalizing behaviors to self-manage their depression symptoms [70].

Some core concepts of TMI are also in direct conflict with help-seeking behavior. For example, seeking help for mental health issues implies a loss of status and requires men to face their vulnerability [27,71,72]. Concordantly, men with high TMI and CMN exhibit more negative attitudes toward help-seeking [[73], [74], [75]], lower willingness to seek help for mental health issues [76], and lower actual help-seeking behavior, such as psychotherapy use [25,26]. Not seeking help when experiencing psychological distress may therefore be another important factor associated with men's increased risk for suicidal behavior [28].

While externalizing depression symptoms and reduced help-seeking behavior may be partially driving men's increased suicide risk, more direct associations between TMI and STBs have been suggested [77,78]. For example, Coleman [79] reported TMI to be a direct risk factor for suicidal ideation among men. A recent study by Coleman et al. [4] even found men with strong TMI to be about 2.4 times more likely to die by suicide, but 1.5 times less likely to report suicidal ideation. Walther et al. [33] found men with experienced status loss – status being a concept rooted in many conceptualizations of TMI – to be more than twice as likely to report suicidal ideation and more than four times more likely to have attempted suicide. Furthermore, TMI related to self-reliance and restrictive emotionality have been shown to be directly associated with men's increased risk for STBs [[29], [30], [31], [32]].

As a potential explanation, Tryggvadottir et al. [35] have proposed that men with strong TMI who experience psychological distress may perceive this state as being incompatible with many core concepts of TMI. More specifically, living in an environment that fosters the need to conform to restrictive TMI may lead some men to see suicide as the only viable way out of their distressed and painful state [17,35,80,81]. Indeed, multiple studies previously reported that suicide was seen by some men as a courageous or masculine attempt to regain control over feelings of being trapped [72,82,83].

### 1.3 Aim of the present study

Taken together, men are more likely to die by suicide than women and, at the same time, less likely to disclose suicidal ideation. Recently, novel risk factors arising from conformity to restrictive TMI have been suggested to play an important role in the understanding of men's increased suicide risk. The present study, as a first of its kind, will use a person-centric approach aimed at disentangling the complex and heterogeneous interplay between CMN, depression, help-seeking, STBs, and suicidogenic beliefs. To this end, the present study will try to answer the following two research questions (RQs).

• RQ1: Are different CMN profiles associated with depression and psychotherapy use?
• RQ2: Are different CMN profiles associated with STB history or intensity of suicidogenic beliefs?

## 2 Material and methods

### 2.1 Design

Data for this study was obtained through an anonymous online survey called *Andromind Selbsttest (“Andromind self-test”; AST)* that was part of a larger research project for men's mental health at the Department of Clinical Psychology and Psychotherapy at the University of Zurich. The majority of participants were recruited through social media advertisements that were geo-restricted to the German-speaking parts of Europe. The recruitment process and data collection started in October 2021 and ended in June 2022, while formal data analysis was conducted in the Fall Semester of 2022. The research project was approved by the ethical review board of the faculty of Arts and Social Sciences of the University of Zurich (approval 21.4.22). The pre-registered hypotheses, analysis plan, and data used for the present study are publicly available under: https://osf.io/vt5s7/[DOI: 10.17605/OSF.IO/VT5S7].

### 2.2 Sample

**Fig. 1:**
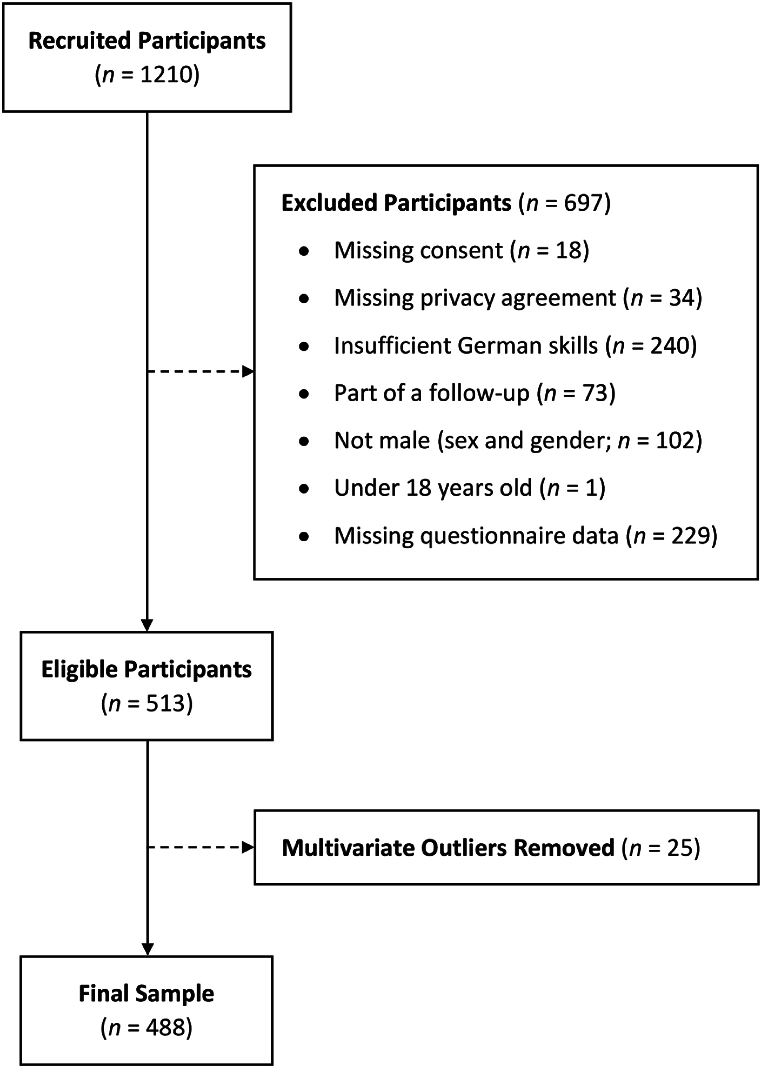
Exclusion Criteria and Participant Flow *Note. n* = number of participants; MRNI-SF = Male Role Norms Inventory – Short Form; CMNI-30 = Conformity to Masculine Norms Inventory – 30.

Out of the 1210 participants recruited for this study, a total of 697 participants were excluded due to one of the following reasons: missing consent or privacy agreement, insufficient German language skills, being part of the follow-up, not self-identifying as a cisgender man, being under 18 years old, or missing data in any of the questionnaires needed for the present study (Fig. 1). Because LPA is highly susceptible to outliers [84], *n* = 25 participants were excluded due to multivariate outliers on the questionnaire used for the LPA. This led to the inclusion of 488 cisgender male participants. All analyses were sufficiently powered to detect at least medium-sized effects (Supplemental Text S1).

### 2.3 Instruments

#### 2.3.1 Sociodemographic Information

Participants were asked to specify their current gender identity as well as the sex they were assigned at birth. Further questions assessed participants' age in years, gross annual household income in Swiss Francs (conversion rate of 1.00 Euro to 1.11 Swiss Francs), highest completed educational attainment, relationship status, self-identified sexual orientation, and their nationality. Finally, participants were asked if they were currently suffering from any formally diagnosed mental disorder (and, if so, what kind of diagnosis) as well as if they were currently receiving psychotherapeutic treatment. A detailed overview of these sociodemographic questions and the respective groupings used for the analyses can be found in Supplemental Table S1.

#### 2.3.2 Traditional masculinity

**Conformity to Masculine Norms Inventory – 30 (CMNI-30).** The CMNI-30 [85] consists of 30 items measuring conformity to masculine norms (CMN). Each item consists of a statement about CMN, such as “I love it when men are in charge of women”, to which participants have to indicate their level of agreement on a six-point Likert scale ranging from 0 = *strongly disagree* to 5 = *strongly agree*. CMN can be divided into ten subscales, namely *Emotional Control*, *Winning*, *Playboy*, *Violence*, *Heterosexual Self-Presentation* (“Heterosexuality”), *Pursuit of Status* (“Status”), *Primacy of Work* (“Work”)*, Power over Women* (“Patriarchic”), *Self-Reliance*, and *Risk-Taking.* The German-language version used in the present study showed satisfactory internal consistencies for all scales (ω > .70), except for the Status scale (ω = .63; [86]). In the present sample, satisfactory internal consistencies were found for all scales except for the Violence scale (Table S2).

#### 2.3.3 Depressive symptoms

**Patient Health Questionnaire – 9 (PHQ-9).** The PHQ-9 [87] consists of nine items assessing prototypical depression symptoms as defined by the DSM-5 [88]. For each item, participants have to indicate how often they were affected by a depression symptom in the past two weeks, for example “Feeling down, depressed, or hopeless”. Answers are possible on a four-point Likert scale ranging from 0 = *not at all* to 3 = *almost every day*. The German version used in the present study showed good internal consistency in the German general population (α = .87; [89]). In the present sample, good internal consistencies were found (Table S2).

**Male Depression Risk Scale – 22 (MDRS-22).** The MDRS-22 [24] consists of 22 items assessing male-typical externalizing depression symptoms. Participants are asked about the frequency of experiencing symptoms such as “I suppressed my negative feelings” in the past month. Answers are possible on an eight-point Likert scale ranging from 0 = *not at all (0 days)* to 7 = *almost always (25+ days)*. The MDRS-22 assesses male-typical externalizing depression symptoms as a general factor and on six subscales, namely *Emotion Suppression*, *Drug Use*, *Alcohol Use*, *Anger and Aggression*, *Somatic Symptoms*, and *Risk-Taking*. The validated German version used in the present study showed at least satisfactory internal consistency in a large German-speaking sample with Guttman's λ_2_ ranging from .77 (Somatic Symptoms) to .91 (Drug Use), except for the Risk-Taking subscale with λ_2_ = .62 [90]. In the present sample, satisfactory internal consistencies were found for all subscales, except for the Risk-Taking subscale (Table S2).

#### 2.3.4 Suicidal thoughts, behaviors and belief systems

**Suicide Ideation and Behavior Scale (SIBS).** The SIBS [91] consists of nine items, six of which assess the frequency of suicidal ideation, suicidal intentions, suicidal impulses, and preparation and planning behavior during the past four weeks. For example, participants are asked how often they " … thought that it would be better if I were not alive”, with answers being possible on a six-point Likert scale ranging from 0 = *never* to 5 = *every day, multiple times*. The questionnaire further includes two dichotomous *Yes/No* questions about past-month suicide attempts and lifetime suicide attempts. A final question asks how often participants attempted suicide (open answer format). The version of the SIBS used in the present study showed good internal consistencies (α = .92; ω = .93) across five independent clinical and non-clinical samples [91]. The present sample showed good internal consistencies (Table S2).

**Suicide Cognitions Scale – 18 (SCS-18).** The SCS-18 [49,51] consists of 18 items that measure central aspects of the suicidal belief system on the three subscales *Unsolvability* (i.e., perceiving an incapability of solving one's problems, with suicide as the only solution), *Unbearability* (i.e., perceiving an incapability to tolerate one's emotional pain), and *Unlovability* (i.e., perceiving oneself as a burden, unworthy of love, or deserving of punishment). Participants have to indicate how strongly they agree with each item (e.g., “I am completely unworthy of love”) on a five-point Likert scale ranging from 1 = *strongly disagree* to 5 = *strongly agree*. The German version used in the present study showed good validity and internal consistencies (total scale: α = .94; Unsolvability: α = .88; Unbearability: α = .88; Unlovability: α = .88) in German outpatient and inpatient samples [50]. In the present sample, good internal consistencies were found (Table S2).

#### 2.3.5 Social desirability

**Marlowe-Crowne Social Desirability Scale (MC-SDS).** The short form of the MC-SDS [92,93] consists of 10 items that assess participants' social desirability in their response style. Participants are asked if they would describe themselves with socially desirable traits (e.g., “I have never intensely disliked anyone”) with a binary response format (0 = *No*, 1 = *Yes*), where higher values in the final sum score indicate stronger social desirability. While the English-language version of the MC-SDS showed a moderate composite reliability of ρx = .83 [93], no German-language version of this questionnaire has been validated so far. The present study therefore used a forward-translated version of the English-language version, which did not show satisfactory internal consistency estimates (Table S2).

### 2.4 Statistical analysis

Prior to the main analysis, the sample adequacy was assessed by screening for multivariate outliers using the Mahalanobis distance [94] and conducting a post-hoc power analysis. Additionally, psychometric properties of the questionnaires (Cronbach's α and McDonald's ω) and potential deviations from the Gaussian distribution (|skewness| > 2 or |kurtosis| > 7; [95]) were assessed. Subsequently, descriptive statistics stratified by lifetime suicide attempts and correlation coefficients for the relevant study variables were calculated. To then answer the proposed research questions, the main analysis consisted of the two parts described in the following.

In a first part, CMN subpopulations (i.e., profiles) were estimated from the CMNI-30 scales using latent profile analysis (LPA). LPA is a model-based clustering approach to model a latent categorical variable from a set of manifest indicator variables through probabilistic classification [96,97]. To ensure parsimony, only models with a maximum of five profiles as well as equal variances and covariances constricted to zero were considered. The optimal number of latent profiles was determined through an analytic hierarchy process [98] and bootstrapped likelihood ratio tests (BLRT; [99]).

In a second part, results from the LPA were used to examine potential associations between individual CMN profiles and different outcome variables. To this end, pairwise χ^2^-, *t*-, and Wilcoxon rank-sum tests were applied to compare the obtained profiles in relation to sociodemographic variables (age, income, education, relationship status, and sexual orientation), depression diagnosis, depressive symptoms (PHQ-9 and MDRS-22), and STBs (SIBS and SCS-18). Lastly, the conditional effect of profile membership on lifetime suicide attempt was estimated with hierarchical binomial logistic regression analyses under consideration of relevant covariates. Standardized effect sizes were computed for all analyses according to the overview provided in Supplemental Table S3.

For all inferential analyses, an initial alpha level of .05 was used to test for statistical significance, followed by a correction for multiple testing according to the Holm-method [100]. All computations were performed with the statistical software *R* (version 4.2.0–2; R Core Team, 2020) and the additional packages *mclust* (version 5; [101]), *psych* [102], *rcompanion* [103], *car* [104], *pwr* [105], and *ggpplot2* [106].

## 3 Results

### 3.1 Descriptive statistics and correlations

Out of the 488 men included in the present sample, 65 men (13.3 %) reported a lifetime suicide attempt, 120 men (24.6 %) reported being formally diagnosed with depression, and 99 men (20.3 %) reported currently using psychotherapy. Men with a suicide attempt were disproportionately often diagnosed with depression, showed stronger CMN, higher levels of depression symptoms, more STBs, and stronger suicidal belief systems (Table 1). Being diagnosed with depression and higher levels of depressive symptoms were linked to psychotherapy use, more STBs, and stronger suicidal belief systems. Stronger CMN was associated with higher levels of depressive symptoms, more STBs, and stronger suicidal belief systems (Table 2).

**Table 1:** Descriptive statistics of the sample stratified by lifetime suicide attempt.

**Variable**	**Total** (*n* = 488)	**Lifetime suicide attempt**			Effect size (*d*/*V*)		95%-CI	
		No (*n* = 423)	Yes (*n* = 65)					
**Age**, *mean (SD)*	44.3 (15.3)	43.9 (15.3)	47.1 (14.8)	−1.61 (86)	.21		[-.05, .47]	
**Nationality**, *n (%)*				4.71 (4)	.10		[.03, .14]	
Swiss	102 (20.9)	93 (22.0)	9 (13.8)					
German	339 (69.5)	288 (68.1)	51 (78.5)					
Austrian	34 (7.0)	29 (6.9)	5 (7.7)					
Luxembourger	1 (.2)	1 (.2)	–					
Other	12 (2.5)	12 (2.8)	–					
**Yearly Income in CHF**, *n (%)*				10.78 (2)	**.15**^a^	small	[.05, .23]	
< 25,000	143 (29.3)	113 (26.7)	30 (46.2)					
25,000–75,000	185 (37.9)	164 (38.8)	21 (32.3)					
> 75,000	160 (32.8)	146 (34.5)	14 (21.5)					
**Highest Education**, *n (%)*				3.50 (4)	.08		[-.01, .15]	
None completed	1 (.2)	1 (.2)	–					
Secondary education	244 (50.0)	206 (48.7)	38 (58.5)					
Tertiary education	215 (44.1)	193 (45.6)	22 (33.8)					
Other	28 (5.7)	23 (5.4)	5 (7.7)					
**In a Relationship**, *n (%)*				5.46 (2)	.11		[.00, .18]	
Yes	234 (48.0)	211 (49.9)	23 (35.4)					
Yes, non-exclusive	33 (6.8)	26 (6.1)	7 (10.8)					
No	221 (45.3)	186 (44.0)	35 (53.8)					
**Sexual Orientation**, *n (%)*				15.25 (5)	**.18**^a^	small	[.05, .23]	
Heterosexual	380 (77.9)	333 (78.7)	47 (72.3)					
Gay	59 (12.1)	54 (12.8)	5 (7.7)					
Bisexual	35 (7.2)	25 (5.9)	10 (15.4)					
Asexual	8 (1.6)	0 (0)	1 (1.5)					
Other	1 (.2)	4 (.9)	1 (1.5)					
Not sure/no answer	5 (1.0)	7 (1.7)	1 (1.5)					
**Mental Health**, *n (%)*								
Depression Diagnosis	120 (24.6)	93 (22.0)	27 (41.5)	10.59 (1)	**.15**^b^	small	[.06, .25]	
Psychotherapy Use	99 (20.3)	80 (18.9)	19 (29.2)	3.10 (1)	.09		[-.01, .18]	
**Questionnaires**, *mean (SD)*								
CMNI-30	51.8 (16.4)	50.9 (15.7)	57.7 (19.6)	−2.64 (77)	**.41**^a^	medium	[.15, .68]	
PHQ-9	9.9 (6.6)	9.1 (6.1)	14.9 (7.3)	−6.11 (78)	**.93**^c^	large	[.66, 1.20]	
MDRS-22	23.0 (18.7)	21.0 (16.3)	36.1 (26.6)	−4.44 (72)	**.84**^c^	large	[.57, 1.11]	
SIBS	1.8 (4.3)	1.1 (2.5)	6.7 (8.6)	−5.21 (66)	**1.44**^c^	large	[1.16, 1.72]	
SCS-18	32.3 (14.9)	30.0 (12.7)	47.3 (19.2)	−7.07 (73)	**1.27**^c^	large	[.99, 1.54]	
Unsolvability	9.7 (4.7)	8.9 (3.7)	14.8 (6.9)	−6.75 (70)	**1.40**^c^	large	[1.12, 1.67]	
Unbearability	12.0 (6.3)	11.2 (5.7)	17.5 (7.3)	−6.61 (76)	**1.06**^c^	large	[.78, 1.33]	
Unlovability	10.6 (5.3)	9.9 (4.6)	15.0 (6.9)	−5.82 (73)	**1.04**^c^	large	[.77, 1.31]	
MC-SDS	4.4 (2.2)	4.5 (2.2)	3.7 (2.1)	2.80 (88)	**−.36**^a^	medium	[-.62, −.10]	

Note. *p*-values were adjusted for multiple testing using the Holm-method while bootstrapped 95 % confidence intervals (95 % CI) are unadjusted.*SD* = standard deviation; *n* = number of participants; *t* = *t*-statistic; χ^2^ = chi-squared-statistic; df = degrees of freedom; *d* = Cohen's *d*; *V* = Cramer's *V*; 95%-CI = bootstrapped 95 % confidence interval for the respective effect; CMNI-30 = Conformity to Masculine Norms Inventory – 30; PHQ-9 = Patient Health Questionnaire – 9; MDRS-22 = Male Depression Risk Scale – 22; SIBS = Suicide Ideation and Behavior Scale; SCS-18 = Suicide Cognition Scale – 18; MC-SDS = Marlowe–Crowne Social Desirability Scale.

a *p* < .05.

b *p* < .01.

c *p* < .001.

**Table 2:** Pearson's Bivariate and point-biserial correlation coefficients for relevant variables.

**Variable**	1	2	3	4	5	6	7	8	9	10	11	12	12.1	12.2	12.3
1. Age (in years)	–														
2. Income (in CHF)	.01	–													
3. Tertiary Education^1^	**.28**^c^	.10	–												
4. In a Relationship^1^	**.16**^a^	.06	.12	–											
5. Heterosexual^1^	.04	−.09	−.01	.01	–										
6. Depression Diagnosis^1^	.03	−.06	−.09	−.15	−.05	–									
7. Psychotherapy Use^1^	−.08	.07	.03	−.09	−.05	**.48**^c^	–								
8. CMNI-30	−.15	.07	−.01	−.09	.09	.01	.07	–							
9. PHQ-9	−.13	−.02	−.10	**−.29**^c^	−.02	**.41**^c^	**.34**^c^	**.25**^c^	–						
10. MDRS-22	−.14	−.06	−.08	**−.17**^a^	−.01	**.27**^c^	**.21**^c^	**.35**^c^	**.71**^c^	–					
11. SIBS	−.03	−.09	−.15	**−.16**^a^	−.05	**.28**^c^	**.22**^c^	**.18**^b^	**.52**^c^	**.45**^c^	–				
12. SCS-18	−.08	−.08	−.12	**−.27**^c^	−.01	**.38**^c^	**.30**^c^	**.24**^c^	**.73**^c^	**.59**^c^	**.64**^c^	–			
12.1 Unsolvability	−.03	−.08	−.11	**−.23**^c^	−.01	**.31**^c^	**.23**^c^	**.26**^c^	**.62**^c^	**.51**^c^	**.71**^c^	**.92**^c^	–		
12.2 Unbearability	−.10	−.06	−.10	**−.26**^c^	−.01	**.42**^c^	**.34**^c^	**.19**^b^	**.74**^c^	**.62**^c^	**.54**^c^	**.92**^c^	**.77**^c^	–	
12.3 Unlovability	−.08	−.09	−.13	**−.26**^c^	−.02	**.30**^c^	**.24**^c^	**.22**^c^	**.63**^c^	**.48**^c^	**.53**^c^	**.91**^c^	**.79**^c^	**.73**^c^	–
13. MC-SDS	.09	−.02	.01	.05	−.03	−.10	−.08	**−.37**^c^	**−.22**^c^	**−.24**^c^	−.12	**−.18**^b^	**−.16**^a^	**−.18**^b^	**−.16**^a^

*Note. p*-values were adjusted for multiple testing using the Holm-method.

CMNI-30 = Conformity to Masculine Norms Inventory – 30; PHQ-9 = Patient Health Questionnaire – 9; MDRS-22 = Male Depression Risk Scale – 22; SIBS = Suicide Ideation and Behavior Scale; SCS-18 = Suicide Cognition Scale – 18; MC-SDS = Marlowe–Crowne Social Desirability Scale. ^1^ point-biserial correlation coefficients were estimated for dichotomized variables.

a *p* < .05.

b *p* < .01.

c *p* < .001.

### 3.2 Latent profile analysis

**Fig. 2:**
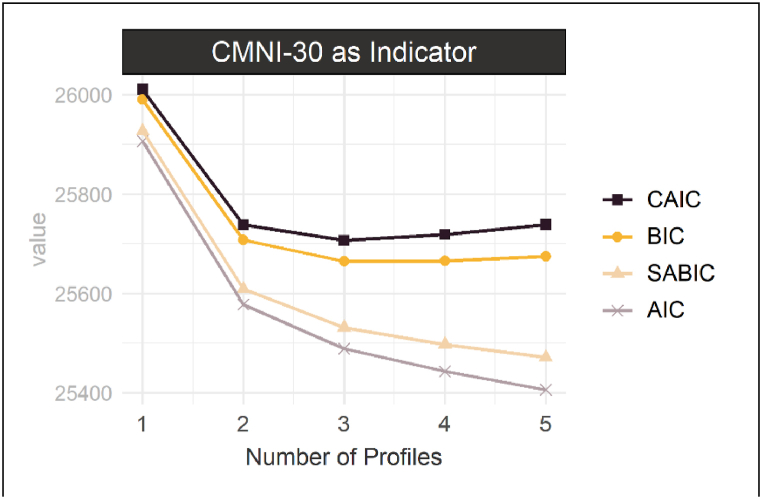
Information Criteria for 1–5 Latent Profile Models *Note.* Information criteria used to determine the number of latent profiles. Lower values indicate a lower prediction error and better model fit. CAIC = consistent Akaike Information Criterion; BIC = Bayesian Information Criterion; AIC = Akaike Information Criterion; SABIC = sample-size adjusted Bayesian Information Criterion.

**Fig. 3:**
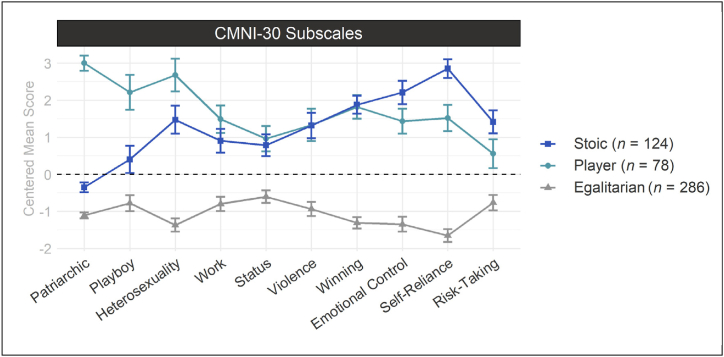
Latent Profile-Derived Subgroups of Men Conforming to TMI *Note.* Centered mean scores for the estimated profiles on the susbcales of the Conformity to Masculine Norms Inventory (CMNI-30). Vertical error bars indicate ±1 standard error. *n* = number of participants.

Latent profile analysis indicated three potential CMN subgroups (i.e., profiles) to be present in the data (Fig. 2, Supplemental Table S4). The largest profile corresponded to a subgroup with overall low CMN (Fig. 3). This subgroup was labeled *Egalitarian(s)* and consisted of 286 men (58.6 %). The second profile was characterized by strong CMN on the dimensions Patriarchic (i.e., men having power, especially over women), Playboy (i.e., endorsing sexual promiscuity), and Heterosexism (i.e., the importance of appearing heterosexual). This second subgroup was labeled *Player(s)* and consisted of 78 men (16.0 %). A third profile was characterized by strong CMN on the dimensions Emotional Control (i.e., needing to have control over one's own emotions), Self-Reliance (i.e., unwillingness to ask for help but rather rely on oneself), and Risk-Taking (i.e., willingly exposing oneself to risky situations). This subgroup was labeled *Stoic(s)* and consisted of the 124 remaining men (25.4 %). A more detailed description of the analysis can be found in the supplementary (Supplemental Text S2).

### 3.3 Subgroup comparisons

**Fig. 4:**
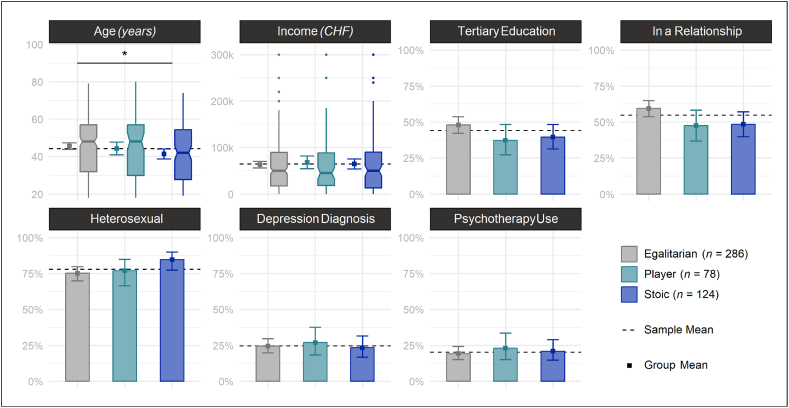
Pairwise Subgroup Comparisons of Sociodemographic and Mental Health Related Variables *Note.* Group differences in sociodemographic variables, depression diagnosis, and psychotherapy use. *p*-values were adjusted for multiple testing using the Holm-method while 95 % Wald confidence intervals (vertical error bars around the mean and notches around the median) are unadjusted. *n* = number of participants.∗*p* < .05; ∗∗∗*p* < .001.

**Fig. 5:**
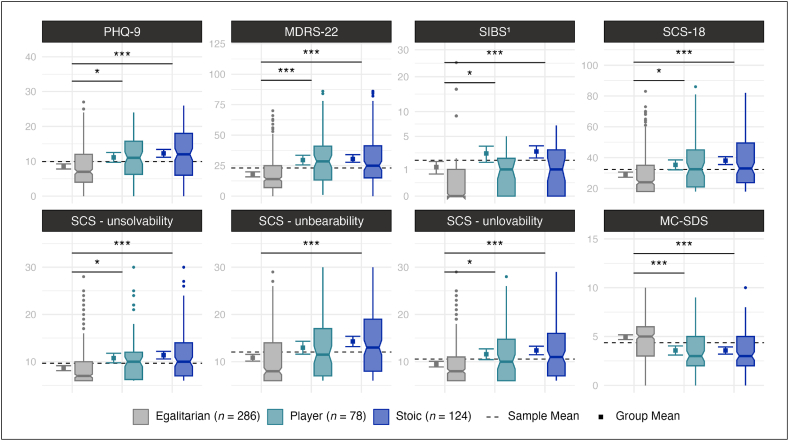
*Pairwise Subgroup Comparisons of Depressive Symptoms, Suicidality, and Social Desirability* *Note*. Group differences in prototypical depression symptoms (PHQ-9), externalizing depression symptoms (MDRS-22), suicide ideation and behavior (SIBS), suicidal belief systems (SCS-18), and social desirability (MC-SDS). *p*-values were adjusted for multiple testing using the Holm-method while 95 % Wald confidence intervals (vertical error bars around the mean and notches around the median) are unadjusted. *n* = number of participants.^1^ non-parametric Wilcoxon rank-sum test was used. ∗*p* < .05, ∗∗∗*p* < .001.

Pairwise comparisons among the CMN subgroups revealed that Stoics were younger than Egalitarians (Fig. 4; Supplemental Table S5), while no statistically significant differences were found regarding income, educational level, relationship status, sexual orientation, depression diagnosis, nor psychotherapy use. Compared to Egalitarians, Players and Stoics showed overall higher levels of prototypical and externalizing depression symptoms (Fig. 5; Supplemental Table S6), as well as more Emotion Suppression and Anger (Fig. S1; Supplemental Table S7). Notably, only Stoics but not Players showed higher levels of Somatic Symptoms and Risk-Taking than Egalitarians. Players and Stoics also showed more STBs, and stronger Unsolvability and Unlovability beliefs than Egalitarians. Importantly, only Stoics but not Players showed stronger Unbearability beliefs than Egalitarians.

### 3.4 Regression analysis

**Fig. 6:**
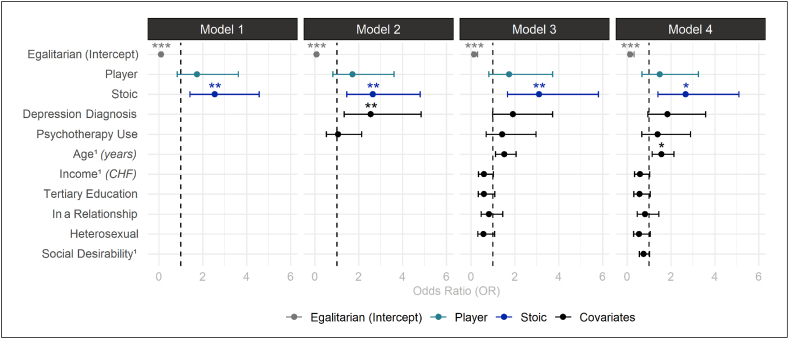
Odds Ratios of Hierarchic Regression Models 1–4 for Lifetime Suicide Attempt *Note.* Reference level is the Egalitarian subgroup without a depression diagnosis, not using psychotherapy, average age and income, no tertiary education, not in a relationship, non-heterosexual, and average social desirability (MC-SDS). *p*-values were adjusted for multiple testing using the Holm-method while 95 % Wald confidence intervals (95 % CI) are unadjusted. *n* = number of participants.^1^ variable was *z-*standardized. ∗*p* < .05; ∗∗*p* < .01; ∗∗∗*p* < .001.

Regression models were used to estimate the association between CMN profile membership and lifetime suicide attempt while controlling for sociodemographic and mental health variables (Fig. 6; Supplemental Table S8). Egalitarians in the reference category showed odds of .15 for a lifetime suicide attempt (=base-rate risk of 9.1 %; [107]). In comparison, Stoics had a 2.32 times higher risk for a lifetime suicide attempt than Egalitarians (= overall risk of 21.1 %). Comparable risks for a lifetime suicide attempt were found between Egalitarians and Players and between Players and Stoics. Comparing the regression models with likelihood ratio tests showed a statistically significant improvement of the model fit up to model 3.

## 4 Discussion

As a first of its kind, the present study examined men's increased suicide risk using a person-centric perspective on the associations between CMN, depression, help-seeking, STBs, and suicidogenic beliefs. Latent profile analysis (LPA) revealed three distinct subgroups among 488 cisgender men taking part in an anonymous cross-sectional online survey. One subgroup was defined by strong CMN about Emotional Control, Self-Reliance, and Risk-Taking (the Stoics; *n* = 124; 25.4 %). Binomial logistic regression analyses showed Stoics to have a 2.32 times higher risk for a lifetime suicide attempt, while also being characterized by younger age, stronger somatization of depressive symptoms, and stronger suicidal beliefs about the unbearability of emotional pain when compared to the majority subgroup defined by overall low CMN (the Egalitarians; *n* = 286; 58.6 %).

### 4.1 Key characteristics of CMN profiles

Among the three analytically derived subgroups of men conforming to TMI, two profiles were characterized by pronounced conformity to specific TMI dimensions. The Stoic's profile, defined by restrictive emotionality, self-reliance, and risky behavior, may be traced back to Robert Brannon's [108] seminal work analyzing the American culture's “blueprint” of masculinity, which informs many modern conceptualizations and measures of masculinity [19]. Brannon identified four pivotal themes of masculinity, one of which – the “sturdy oak” – was centered around men needing to be tough and not showing weaknesses [109]. The Player's profile, on the other hand, revolves around societal dominance and power, which may be traced back to feminist theories about patriarchal and sexist sociocultural norms in Western societies [20,110,111]. Concordantly, Players in the present sample are defined by conforming to patriarchal and (hetero-)sexist beliefs, as well as their endorsement of sexual promiscuity. Two previous studies using the same methodological approach have identified a conceptually similar CMN profile among adolescent and young male athletes, which was labeled “Jocks” and characterized by a strong focus on (hetero-) sexual prowess and dominance [112,113].

### 4.2 CMN profiles, depression, and psychotherapy use

Regarding depression and psychotherapy use (RQ1), Players and Stoics both showed higher levels of prototypical and externalizing depression symptoms in comparison to Egalitarians, but comparable prevalence rates of formal depression diagnoses and psychotherapy use. Thus, Players and Stoics who experience high levels of depression symptoms appear less likely to be formally diagnosed with depression, less likely to seek psychotherapeutic treatment, and, thus, less likely to receive professional help when experiencing depression symptoms. Notably, two dimensions of externalizing depression symptoms were found to characterize Stoics more than Players. Namely, Stoics showed higher levels of somatic symptoms and risk-taking behavior than Egalitarians, which was not the case for Players. Somatic symptoms play an important role in the *masked depression framework* [22], which assumes that certain men mask prototypical depression symptoms such as sadness, grief, or vulnerability, by experiencing or expressing externalizing symptoms that are more in line with conforming to TMI [27,63]. Thus, pronounced somatization of depression symptoms may prevent men from receiving – rather than seeking – the help they need, even though they were initially willing to seek help, in this case most likely from a general practitioner. Risk-taking behavior, on the other hand, seems to be primarily attributable to the strong expression of the CMNI-30's Risk-Taking dimension in the Stoic's profile.

### 4.3 CMN profiles and STB

Regarding STBs (RQ2), the most pivotal findings of this study are the 2.32 times higher risk for a lifetime suicide attempt among Stoics and their stronger suicidal beliefs about the unbearability of their emotional pain when compared to Egalitarians. Players, on the other hand, did not show an increased risk for a lifetime suicide attempt nor stronger suicidal unbearability beliefs. Consequently, the Stoics' specific constellation of CMN, namely restrictive emotionality, being self-reliant, and more willingly engaging in risky behavior, paired with a pronounced suicidal belief system about one's emotional pain being unbearable, seems to describe a subtype of men that is highly vulnerable to engage in suicidal behavior. While individual CMN dimensions underlying the Stoic's profile have previously been empirically related to increased risk for STBs among men (e.g., self-reliance and restrictive emotionality; [31,32]), the specific CMN constellation identified in the present sample may be indicative of a broader psychosocial predicament that further elevates these men's risk for a suicide attempt.

For example, Cleary [17] proposed a psychosocial framework in which distressed men who live in environments that foster hegemonic TMI are restricted from expressing their emotional pain or reaching out for help, which greatly narrows their options to effectively deal with that pain. Combined with a suicidal belief system that propagates the unbearability of their pain and the situation in which they find themselves in, suicide may become their only way out. If Stoics nonetheless reach out and seek professional help at some point, their proneness to externalizing depression symptoms as somatic symptoms or risk-taking behavior may leave their underlying depressive symptoms unrecognized by healthcare professionals [[114], [115], [116]]. Concordantly, Schaffer et al. [117] reported that a large portion of men access some form of health care prior to suicide, yet their suicidality was not recognized by providers. However, in light of this study, the possibility also exists that either these men's masked depression symptoms might not have been recognized as such, or their conformity to restrictive TMI could have prevented them from disclosing suicidal ideation or suicide plans [3]. Thus, Stoics may not only be at increased risk for suicidal behavior, but they might also be very challenging to identify as a high-risk subgroup with strong STBs.

### 4.4 Implications

Based on our findings, we recommend tailored intervention programs for men in this high-risk subgroup to encourage open emotional expression, promote and normalize help-seeking behaviors, and provide strategies to mitigate risky behaviors to reduce their risk of suicidal behaviors. Furthermore, fostering alternative perspectives on traditional masculinity, such as the expectation to provide for and protect one's family, may serve as a protective factor against suicidal behavior [118]. Lastly, we strongly advocate for integrating considerations about CMN into existing mental health policies and programs to ensure they are sensitive to gender norms. This approach has been successfully implemented by programs such as *Men in Mind* for healthcare practitioners [119] or *Heads Up Guys!* targeted specifically at men [120].

### 4.5 Limitations

Some important limitations must be considered when interpreting our findings. Overall, the study's sample consisted of a large portion of highly psychologically distressed men, as evidenced by the high prevalence for a lifetime suicide attempt (13.3 %) and formal depression diagnoses (24.4 %), limiting generalizability to broader and less distressed population samples. From a methodological perspective, even though the study's sample is substantial (488 men, nearing the 500 proposed by Spurk et al. [121] for LPA), and despite the strong restrictions to increase parsimony (i.e., maximum of five profiles, equal variances across profiles, and trivial covariance), not all fit indices converged toward a singular solution. Furthermore, the overall trend between stronger CMN and more negative outcomes (e.g., depression or STBs) irrespective of profile membership, suggests that the assumed underlying mixture distributions may not fulfill the local independence assumption of LPA [121]. Due to the limitations of the present study, the theoretical and practical effects of our findings require further investigation in future research.

### 4.6 Future directions

We suggest the following recommendations for future research into this topic. First, the results of this study need to be replicated in a sample of men who are less psychologically distressed, ideally being more representative of the male general population. Particularly considering the ongoing discussion about the measurement invariance of the CMNI-30 (e.g., Refs. [122,123]), a geographically more diverse sample may allow for a more fine-grained interpretation of results. Second, the present findings may be examined in a longitudinal context. Despite indications of TMI constructs [124] and suicidal beliefs [50] being somewhat time-stable, intraindividual changes could still provide important insights into the underlying dynamics of these constructs. Third, future studies may try to replicate our findings using a factor-analytic approach which does not rely on local independence assumptions. Fourth, it might be useful to try and disentangle the underlying processes in future studies using real-time or idiographic approaches.

## 5 Conclusion

To conclude, the analytically derived profile of the Stoic, defined by strong CMN about restrictive emotionality, self-reliance, and risk-taking, showed an increased risk for a lifetime suicide attempt, stronger suicidal beliefs about the unbearability of emotional pain, stronger externalizing depression symptoms related to somatization and risk-taking behavior. These findings can potentially help to understand how a specific subgroup of men, entrenched in restrictive socialized gender norms, might find themselves in a situation where they perceive suicide as the only viable way out of their emotional pain. However, the present results warrant replications in more representative male population samples.

## CRediT authorship contribution statement

**Lukas Eggenberger:** Writing – review & editing, Writing – original draft, Visualization, Validation, Methodology, Investigation, Formal analysis, Data curation, Conceptualization. **Lena Spangenberg:** Writing – review & editing, Investigation. **Matthew C. Genuchi:** Writing – review & editing, Investigation. **Andreas Walther:** Writing – review & editing, Validation, Supervision, Resources, Project administration, Methodology, Investigation, Funding acquisition, Conceptualization.

## Informed consent

Informed consent was obtained from all subjects involved in the study.

## Data availability

The pre-registered hypotheses, analysis plan, and data used for the present study are publicly available under: https://osf.io/vt5s7/[DOI: 10.17605/OSF.IO/VT5S7].

## Ethical approval

The study was conducted according to the guidelines of the Declaration of Helsinki and the study was approved by the ethical committee of the Faculty of Arts and Social Sciences of the University of Zurich (21.4.22).

## Funding

This study was funded by a grant from the 10.13039/100000001Swiss National Science Foundation awarded to AW (PZPGP1_201757).

## Declaration of competing interest

The authors declare that they have no known competing financial interests or personal relationships that could have appeared to influence the work reported in this paper.
