# Supplementary material for: Men's Suicidal thoughts and behaviors and conformity to masculine norms: A person-centered, latent profile approach
Source: Heliyon. 2024 Oct 9;10(20):e39094. doi: 10.1016/j.heliyon.2024.e39094 (PMC11620066; doi:10.1016/j.heliyon.2024.e39094)
Supplement: Multimedia component 1 [file mmc1.docx]

**Men's Suicidal Thoughts and Behaviors and Conformity to Masculine Norms:
A Person-Centered, Latent Profile Approach**

**Supplementary Materials**

Lukas Eggenberger^1,2^, Lena Spangenberg^3^, Matthew C. Genuchi^4^, Andreas Walther^5,*^

^1^ Experimental and Clinical Pharmacopsychology, Department of Adult Psychiatry and Psychotherapy, University Hospital of Psychiatry Zurich and University of Zurich, Zurich, Switzerland

^2^ Jacobs Center for Productive Youth Development, University of Zurich, Zurich, Switzerland

^3^ Department of Medical Psychology and Medical Sociology, University Leipzig, Leipzig, Germany

^4^ Department of Psychological Science, Boise State University, Boise, Idaho, USA

^5^ Clinical Psychology and Psychotherapy, Psychological Institute, University of Zurich, Zurich, Switzerland

***Corresponding author**: Dr. Andreas Walther, Binzmühlestrasse 14, 8050, Zürich; ClinicalPsychology and Psychotherapy, University of Zurich, Zurich, Switzerland,a.walther@psychologie.uzh.ch

**Text S1***Post-hoc Power Analyses*

Post-hoc power analyses were conducted to determine the minimum number of participants that were needed to detect medium-sized (Cohen's *d* > .50; Cramer's *V* > .30; Cohen's *f*^2^ > .15) effects in each analysis. For the *t*-tests (two-sided; α-level = .05; power = .80), at least *n* = 64 participants per group were needed. For the χ^2^-tests (df = 1; α-level = .05; power = .80), at least *n* = 88 participants in total were needed. For the most extensive multiple regression models, including an intercept, profile membership, and relevant covariates (df_num_ = 10; α-level = .05; power = .80), at least *n* = 118 participants in total were needed. Hence, all analyses were sufficiently powered to detect at least medium-sized effects.

**Text S2**
*Detailed Results of the Latent Profile Analysis*

Information criteria for the latent profile models, conditional on equal variances and covariances constricted to zero, reached a minimum for the CAIC and the BIC at a model with three latent profiles, indicating this model to best fit the underlying data (Figure 2). Importantly, bootstrapped likelihood ratio tests (Supplemental Table S4) reached significant *p*-values for all models, which indicates that the adjacent, more complex model (i.e., one more latent profile) was always more favorable than the more parsimonious model. However, because two out of four information criteria as well as the entropy converged on the model with three latent profiles, this model was assumed to best fit the data in the present sample and thus used for all subsequent analyses.

The largest profile corresponded to a subgroup with an overall low conformity to each individual TMI dimension (Figure 3). Consequently, this subgroup was labeled as *Egalitarian(s)*, consisting of a total of 286 men (58.6%). The second profile was characterized by strong CMN on the dimensions Patriarchic (i.e., men having power, especially over women), Playboy (i.e., endorsing sexual promiscuity), and Heterosexism (i.e., the importance of not appearing non-heterosexual; [74]). This subgroup was labeled *Player(s)*, which was also the smallest profile with only 78 men (16.0%). A third profile was characterized by strong CMN on the dimensions Emotional Control (i.e., needing to have control over one's own emotions), Self-Reliance (i.e., unwillingness to ask for help but rather rely on oneself), and Risk-Taking (i.e., willingly exposing oneself to risky situations; [74]). This subgroup was therefore labeled *Stoic(s)* and consisted of the 124 remaining men (25.4%).

| **Table S1** *Overview of Questions (Q), Answer Options (A), Groupings (Grp.), and Reference Levels (Ref.)* | |
| --- | --- |
| Q*:* | "What is your current gender identity?" |
| A: | 1 = *male*; 2 = *female*; 3 = *female-to-male transgender*; 4 = *male-to-female-transgender;* 5 = *gender neutral / non-binary;* 6 = *diverse*; 7 = *other* (with a free-text option); 8 = *don’t know / not sure* |
| Q: | "What sex were you assigned at birth ('biological sex')?" |
| A: | 1 = *male*; 2 = *female*; 3 = *diverse*; 4 = *other* (with a free-text option); 5 = *don’t know / not sure* |
| Q: | "Please enter your age in years (e.g., 42)." |
| A: | free-text option |
| Q: | "Please enter your annual gross household income in Swiss Francs (CHF; conversion rate of 1 Euro = 1.11 CHF)." |
| A: | free-text option |
| Grp.: | 1 = *below 25,000 CHF*; 2 = *between 25,000 to 75,000 CHF*; 3 = *above 75,000 CHF* |
| Q: | "What is your highest completed education?" |
| A: | 1 = *no completed education*; 2 = *special school*; 3 = *secondary school*; 4 = *high school*; 5 = *basic apprenticeship*; 6 = *apprenticeship*; 7 = *gymnasium*; 8 = *university*; 9 = *other* |
| Grp.: | 1 = *none completed*; 2 = *secondary education*; 3 = *tertiary education*; 4 = *other* |
| Ref.: | 0 = *no tertiary education* |
| Q: | "Are you currently in an intimate relationship?" |
| A: | 1 = *yes*; 2 = *no*; 3 = *yes, but not in an exclusive relationship* |
| Ref.: | 0 = *no intimate relationship* |
| Q: | "What is your sexual orientation?" |
| A: | 1 = *heterosexual*; 2 = *gay / lesbian*; 3 = *bisexual*; 4 = *asexual*; 5 = *other* (with a free-text option); 6 = *don’t know / not sure* |
| Grp.: | 1 = *heterosexual*; 2 = *non-heterosexual* |
| Ref.: | 0 = *non-heterosexual* |
| Q: | "Which nationality do you belong to?" |
| A: | 1 = *Swiss*; 2 = *German*; 3 = *Austrian*; 4 = *Liechtensteiner*; 5 = *Luxembourger*;  6 = *Belgian*; 7 = *other* |
| Q: | "Are you currently suffering from a diagnosed acute or chronic mental disorder?" |
| A: | 1 = *yes*; 2 = *no* |
| Ref.: | 0 = *no diagnosed mental disorder* |
| Q: | "Are you currently receiving psychotherapeutic treatment?" |
| A: | 1 = *yes*; 2 = *no* |
| Ref.: | 0 = *no psychotherapeutic treatment* |
| *Note*. *Q* = translated question; *A* = translated answer options; *Grp.* = groupings used for the description of the sample; *Ref.* = dichotomized reference levels for regression analyses. | |

| **Table S2** *Psychometric Properties of the Questionnaires and Item-Level Mean Scores* | | | | | | | |
| --- | --- | --- | --- | --- | --- | --- | --- |
|  | ***n*_items_** | **α** | **ω** | **Mean (SD)** | **Range** | **Skewness** | **Kurtosis** |
| **CMNI-30** | 30 | .83 | .87 | 1.73 (0.55) | [0; 5] | 0.43 | 0.30 |
| Emotional Control | 3 | .91 | .91 | 2.56 (1.25) |  | 0.13 | -0.71 |
| Winning | 3 | .76 | .78 | 1.95 (1.03) |  | 0.21 | -0.16 |
| Playboy | 3 | .83 | .84 | 1.82 (1.34) |  | 0.47 | -0.56 |
| Violence | 3 | .64 | .64 | 1.49 (1.22) |  | 0.55 | -0.42 |
| Heterosexuality | 3 | .92 | .93 | 1.21 (1.29) |  | 1.01 | 0.33 |
| Status | 3 | .70 | .72 | 2.43 (1.03) |  | 0.02 | -0.10 |
| Work | 3 | .83 | .85 | 1.80 (1.15) |  | 0.33 | -0.47 |
| Patriarchic | 3 | .78 | .79 | 0.67 (0.83) |  | 1.36 | 1.59 |
| Self-Reliance | 3 | .71 | .73 | 1.88 (1.18) |  | 0.10 | -0.83 |
| Risk-Taking | 3 | .89 | .90 | 2.41 (1.20) |  | 0.18 | -0.53 |
| **PHQ-9** | 9 | .90 | .92 | 1.10 (0.73) | [0; 3] | 0.53 | -0.69 |
| **MDRS-22** | 22 | .89 | .92 | 1.05 (0.85) | [0; 7] | 1.40 | 2.84 |
| Emotion Suppression | 4 | .82 | .83 | 2.61 (1.81) |  | 0.45 | -0.78 |
| Drug Use^1^ | 3 | .91 | .92 | 0.38 (1.13) |  | 3.81 | 15.05 |
| Alcohol Use^1^ | 4 | .89 | .91 | 0.75 (1.41) |  | 2.43 | 5.67 |
| Anger/Aggression^1^ | 4 | .88 | .92 | 0.74 (1.07) |  | 2.54 | 7.81 |
| Somatic Symptoms | 4 | .79 | .83 | 0.94 (1.19) |  | 1.95 | 3.98 |
| Risk-Taking^1^ | 3 | .67 | .71 | 0.59 (0.94) |  | 2.79 | 10.32 |
| **SIBS^1^** | 6 | .94 | .96 | 0.30 (0.72) | [0; 6] | 4.13 | 20.62 |
| **SCS-18** | 18 | .96 | .97 | 1.79 (0.83) | [1; 5] | 1.13 | 0.67 |
| Unsolvability | 6 | .89 | .95 | 1.61 (0.78) |  | 1.74 | 2.97 |
| Unbearability | 6 | .93 | .94 | 2.01 (1.05) |  | 0.83 | -0.34 |
| Unlovability | 6 | .90 | .92 | 1.76 (0.88) |  | 1.31 | 1.09 |
| **MC-SDS** | 10 | .60 | .66 | 0.44 (0.22) | [0; 1] | 0.11 | -0.59 |
| Note. *n*_items_ = number of items; α = Cronbach's alpha; ω = McDonald's omega; SD = standard deviation; CMNI-30 = Conformity to Masculine Norms Inventory – 30; PHQ-9 = Patient Health Questionnaire – 9; MDRS-22 = Male Depression Risk Scale – 22; SIBS = Suicide Ideation and Behavior Scale; SCS-18 = Suicide Cognition Scale – 18; MC-SDS = Marlowe–Crowne Social Desirability Scale.  ^1^ identified as non-Gaussian distributions (\|skewness\| > 2; \|kurtosis\| > 7). | | | | | | | |

| **Table S3**  *Standardized Effect Size Measures and Their Interpretation According to Cohen (1988) and Raftery (1995)* | | | |
| --- | --- | --- | --- |
| **Setting** | **Effect Size** | **Thresholds** | **Interpretation** |
|  |  | > 0.20 | small |
| Mean and proportion (2 groups) difference | Cohen's *d*, Cohen's *h* | > 0.50 | medium |
|  |  | > 0.80 | large |
|  |  | > .10 | small |
| Proportion difference (> 2 groups) | Cramer's *V* | > .30 | medium |
|  |  | > .50 | large |
|  |  | > .10 | small |
| Point-, rank-biserial, and Pearson correlation | *r*_point_, *r*_rank_, *r* | > .30 | medium |
|  |  | > .50 | large |
|  |  | > .02 | small |
| Multiple regression (power analysis) | Cohen's *f^2^* | > .15 | medium |
|  |  | > .35 | large |
|  |  | > 1.44 | small |
| Logistic regression | Odds Ratio (OR) | > 2.48 | medium |
|  |  | > 4.27 | large |
|  | | | |

| **Table S4**  *Fit Indices for 1–5 Latent Profile Models of the CMNI-30* | | | | | | | |
| --- | --- | --- | --- | --- | --- | --- | --- |
| Profiles | AIC | CAIC | BIC | SABIC | LR_χ2_ | *p*-value | Entropy |
| n = 1 | 25906.63 | 26010.44 | 25990.44 | 25926.96 | – | – | 1.00 |
| n = 2 | 25577.55 | 25738.45 | 25707.45 | 25609.06 | 351.08 | **.010^**^** | 0.75 |
| n = 3 | 25488.76 | 25706.75 | 25664.75 | 25531.44 | 110.80 | **.010^**^** | 0.72 |
| n = 4 | 25443.67 | 25718.75 | 25665.75 | 25497.53 | 67.09 | **.010^**^** | 0.73 |
| n = 5 | 25406.56 | 25738.74 | 25674.74 | 25471.61 | 59.10 | **.010^**^** | 0.73 |
| *Note.* Fit indices used to determine the number of latent profiles, with lower values indicating a lower prediction error. A non-significant Likelihood ratio chi-squared (LR_χ2_) test would indicate a better fit of the model with one fewer profile, which was not reached. *n* = number of profiles; AIC = Akaike Information Criterion; CAIC = consistent Akaike Information Criterion; BIC = Bayesian Information Croterion; SABIC = sample-size adjusted Bayesian Information Criterion.  ^**^ *p* < .01 | | | | | | | |

| **Table S5**  *Pairwise Subgroup Comparisons of Sociodemographic and Mental Health Related Variables* | | | | | | | |
| --- | --- | --- | --- | --- | --- | --- | --- |
| **Variable**, *mean (SD)* | **Profile Membership** | | | *t* (df) / *U* | Cohen’s *d* / *r*_rank_ | | 95% CI |
|  | Egalitarian (*n* = 286) | Player (*n* = 78) | Stoic (*n* = 124) |  |  |  |  |
| Age | 45.56 (15.23) | 44.35 (15.52) |  | 0.61 (121) | 0.08 |  | [-0.16, 0.34] |
|  | 45.56 (15.23) |  | 41.40 (15.04) | 2.56 (237) | **0.28^*^** | small | [0.06, 0.47] |
|  |  | 44.35 (15.52) | 41.40 (15.04) | 1.33 (160) | 0.19 |  | [-0.10, 0.52] |
| Income^1^ (in 1,000 CHF) | 64.34 (64.93) | 91.42 (17.60) |  | 11291.50 | .01 |  | [.00, .12] |
|  | 64.34 (64.93) |  | 65.35 (62.75) | 17531.00 | .01 |  | [.00, .12] |
|  |  | 91.42 (17.60) | 65.35 (62.75) | 4738.50 | .02 |  | [.00, .17] |
| **Variable**, *n (%)* |  | | | χ^2^ (df) | Cohen’s *h* | |  |
| Tertiary Education | 137 (47.9) | 29 (37.2) |  | 2.42 (1) | 0.22 |  | [-0.18, 0.62] |
|  | 137 (47.9) |  | 49 (39.5) | 2.13 (1) | 0.17 |  | [-0.16, 0.50] |
|  |  | 29 (37.2) | 49 (39.5) | 0.03 (1) | -0.05 |  | [-0.51, 0.41] |
| In a Relationship | 170 (59.4) | 37 (47.4) |  | 3.13 (1) | 0.24 |  | [-0.11, 0.60] |
|  | 170 (59.4) |  | 60 (48.4) | 3.85 (1) | 0.22 |  | [-0.08, 0.53] |
|  |  | 37 (47.4) | 60 (48.4) | 0.00 (1) | -0.02 |  | [-0.41, 0.37] |
| Heterosexual-Identified | 215 (75.2) | 60 (76.9) |  | 0.03 (1) | -0.04 |  | [-0.33, 0.25] |
|  | 215 (75.2) |  | 105 (84.7) | 4.02 (1) | -0.24 |  | [-0.47, -0.01] |
|  |  | 60 (76.9) | 105 (84.7) | 1.44 (1) | -0.20 |  | [-0.51, 0.12] |
| Depression Diagnosis | 70 (24.5) | 21 (26.9) |  | 0.09 (1) | -0.06 |  | [-0.54, 0.43] |
|  | 70 (24.5) |  | 29 (23.4) | 0.01 (1) | 0.03 |  | [-0.41, 0.46] |
|  |  | 21 (26.9) | 29 (23.4) | 0.16 (1) | 0.08 |  | [-0.48, 0.64] |
| Psychotherapy Use | 55 (19.2) | 18 (23.1) |  | 0.35 (1) | -0.09 |  | [-0.63, 0.44] |
|  | 55 (19.2) |  | 26 (21.0) | 0.07 (1) | -0.04 |  | [-0.51, 0.42] |
|  |  | 18 (23.1) | 26 (21.0) | 0.03 (1) | 0.05 |  | [-0.55, 0.65] |
| *Note.* *p*-values were adjusted for multiple testing using the Holm-method while 95% confidence intervals (95% CI) are unadjusted. *n* = number of participants; *t* = *t*-statistic; χ^2^ = chi-squared statistic; *U* = Mann-Whitney-U statistic.  ^1^ non-parametric Wilcoxon rank-sum test was used. ^*^ *p* < .05 | | | | | | | |

| **Table S6**  *Pairwise Subgroup Comparisons of Depressive Symptoms, Suicidality, and Social Desirability* | | | | | | | |
| --- | --- | --- | --- | --- | --- | --- | --- |
| **Questionnaire**, *mean (SD)* | **Profile Membership** | | | *t* (df) / *U* | Cohen’s *d* / *r*_rank_ | | 95% CI |
|  | Egalitarian (*n* = 286) | Player (*n* = 78) | Stoic (*n* = 124) |  |  |  |  |
| PHQ-9 | 8.52 (6.10) | 11.12 (6.19) |  | -3.29 (121) | **-0.42^*^** | small | [-0.68, -0.17] |
|  | 8.52 (6.10) |  | 12.27 (7.00) | -5.18 (208) | **-0.57^***^** | medium | [-0.80, -0.36] |
|  |  | 11.12 (6.19) | 12.27 (7.00) | -1.23 (179) | -0.18 |  | [-0.47, 0.10] |
| MDRS-22 | 17.87 (14.57) | 29.55 (19.24) |  | -4.99 (102) | **-0.68^***^** | medium | [-0.95, -0.41] |
|  | 17.87 (14.57) |  |  | -5.89 (169) | **-0.68^***^** | medium | [-0.88, -0.48] |
|  |  | 29.55 (19.24) |  | -0.45 (183) | -0.06 |  | [-0.33, 0.23] |
| SIBS^1^ | 1.18 (3.25) | 2.55 (5.41) |  | 8791.00 | **.18^*^** |  | [.06, .28] |
|  | 1.18 (3.25) |  | 2.79 (5.39) | 13649.50 | **.21^***^** | small | [.11, .31] |
|  |  | 2.55 (5.41) | 2.79 (5.39) | 4733.50 | .02 |  | [.00, .16] |
| SCS-18 | 28.95 (12.89) | 35.31 (16.17) |  | -3.20 (105) | **-0.43^*^** | small | [-0.68, -0.20] |
|  | 28.95 (12.89) |  | 38.04 (16.44) | -5.47 (192) | **-0.62^***^** | medium | [-0.83, -0.39] |
|  |  | 35.31 (16.17) | 38.04 (16.44) | -1.16 (166) | -0.17 |  | [-0.46, 0.12] |
| Unsolvability | 8.63 (3.91) | 10.77 (5.23) |  | -3.36 (102) | **-0.46^*^** | small | [-0.69, -0.22] |
|  | 8.63 (3.91) |  | 11.39 (5.32) | -5.18 (183) | **-0.59^***^** | medium | [-0.79, -0.39] |
|  |  | 10.77 (5.23) | 11.39 (5.32) | -0.81 (166) | -0.12 |  | [-0.41, 0.14] |
| Unbearability | 10.83 (5.71) | 12.96 (6.51) |  | -2.63 (111) | -0.35 |  | [-0.60, -0.10] |
|  | 10.83 (5.71) |  | 14.28 (6.83) | -4.93 (201) | **-0.55^***^** | medium | [-0.79, -0.33] |
|  |  | 12.96 (6.51) | 14.28 (6.83) | -1.38 (170) | -0.20 |  | [-0.51, 0.06] |
| Unlovability | 9.49 (4.51) | 11.58 (5.68) |  | -2.99 (105) | **-0.41^*^** | small | [-0.66, -0.13] |
|  | 9.49 (4.51) |  | 12.37 (6.03) | -4.77 (185) | **-0.54^***^** | medium | [-0.76, -0.32] |
|  |  | 11.58 (5.68) | 12.37 (6.03) | -0.94 (171) | -0.14 |  | [-0.42, 0.12] |
| MC-SDS | 4.93 (2.15) | 3.56 (1.99) |  | 5.28 (130) | **0.65^***^** | medium | [0.41, 0.93] |
|  | 4.93 (2.15) |  | 3.56 (2.00) | 6.21 (250) | **0.65^***^** | medium | [0.44, 0.88] |
|  |  | 3.56 (1.99) | 3.56 (2.00) | 0.00 (164) | 0.00 |  | [-0.30, 0.27] |
| *Note.* *p*-values were adjusted for multiple testing using the Holm-method while 95% confidence intervals (95% CI) are unadjusted. *n* = number of participants; *t* = *t*-statistic; *U* = Mann-Whitney-U statistic; *r*_rank_ = rank-biserial correlation; PHQ-9 = Patient Health Questionnaire – 9; MDRS-22 = Male Depression Risk Scale – 22; SIBS = Suicide Ideation and Behavior Scale; SCS-18 = Suicide Cognition Scale – 18; MC-SDS = Marlowe–Crowne Social Desirability Scale.  ^1^ non-parametric Wilcoxon rank-sum test was used. ^*^ *p* < .05; ^**^ *p* < .01; ^***^ *p* < .001 | | | | | | | |

| **Table S7**  *Pairwise Subgroup Comparisons of Externalizing Depression Symptoms* | | | | | | | |
| --- | --- | --- | --- | --- | --- | --- | --- |
| **Questionnaire**, *mean (SD)* | **Profile membership** | | | *t* (df) / *U* | Cohen’s *d* / *r*_rank_ | | 95% CI |
|  | Egalitarian (*n* = 286) | Player (*n* = 78) | Stoic (*n* = 124) |  |  |  |  |
| MDRS-22 | 17.87 (14.57) | 29.55 (19.24) |  | -4.99 (102) | **-0.68^***^** | medium | [-0.95, -0.41] |
|  | 17.87 (14.57) |  |  | -5.89 (169) | **-0.68^***^** | medium | [-0.88, -0.48] |
|  |  | 29.55 (19.24) |  | -0.45 (183) | -0.06 |  | [-0.33, 0.23] |
| Emotion Suppression | 8.46 (6.50) | 13.36 (7.93) |  | -5.02 (107) | **-0.68^***^** | medium | [-0.97, -0.42] |
|  | 8.46 (6.50) |  | 13.11 (7.04) | -6.29 (218) | **-0.69^***^** | medium | [-0.91, -0.47] |
|  |  | 13.36 (7.93) | 13.11 (7.04) | 0.22 (149) | 0.03 |  | [-0.24, 0.32] |
| Drug Use^1^ | 0.92 (2.97) | 1.06 (2.71) |  | 10869.50 | .03 |  | [.00, .14] |
|  | 0.92 (2.97) |  | 1.73 (4.47) | 15937.50 | .11 |  | [.02, .22] |
|  |  | 1.06 (2.71) | 1.73 (4.47) | 4465.50 | .09 |  | [.00, .22] |
| Alcohol Use^1^ | 2.36 (4.80) | 3.76 (5.61) |  | 9449.00 | .12 |  | [.02, .22] |
|  | 2.36 (4.80) |  | 3.95 (7.06) | 16624.00 | .05 |  | [.00, .16] |
|  |  | 3.76 (5.61) | 3.95 (7.06) | 5174.00 | .06 |  | [.00, .21] |
| Anger/Aggression^1^ | 2.12 (3.30) | 4.26 (5.71) |  | 8608.00 | **.17^*^** | small | [.06, .27] |
|  | 2.12 (3.30) |  | 4.13 (4.81) | 12247.50 | **.25^***^** | small | [.16, .34] |
|  |  | 4.26 (5.71) | 4.13 (4.81) | 4541.50 | .05 |  | [.00, .20] |
| Somatic Symptoms | 2.88 (3.70) | 4.63 (5.68) |  | -2.57 (96) | -0.36 | small | [-0.59, -0.10] |
|  | 2.88 (3.70) |  | 5.20 (5.75) | -4.13 (169) | **-0.48^***^** | small | [-0.69, -0.29] |
|  |  | 4.63 (5.68) | 5.20 (5.75) | -0.70 (165) | -0.10 |  | [-0.39, 0.17] |
| Risk-Taking^1^ | 1.14 (1.75) | 2.49 (3.38) |  | 8951.50 | .15 | small | [.04, .25] |
|  | 1.14 (1.75) |  | 2.76 (3.84) | 12797.00 | **.23^***^** | small | [.14, .33] |
|  |  | 2.49 (3.38) | 2.76 (3.84) | 4534.50 | .05 |  | [.00, .18] |
| *Note.* *p*-values were adjusted for multiple testing using the Holm-method while 95% confidence intervals (95% CI) are unadjusted. *n* = number of participants; *t* = *t*-statistic; *U* = Mann-Whitney-U statistic; *r*_rank_ = rank-biserial correlation; MDRS-22 = Male Depression Risk Scale – 22.  ^1^ non-parametric Wilcoxon rank-sum test was used. ^*^ *p* < .05; ^***^ *p* < .001 | | | | | | | |

| **Figure S1**  *Pairwise Subgroup Comparisons of Externalizing Depression Symptoms* |
| --- |
| 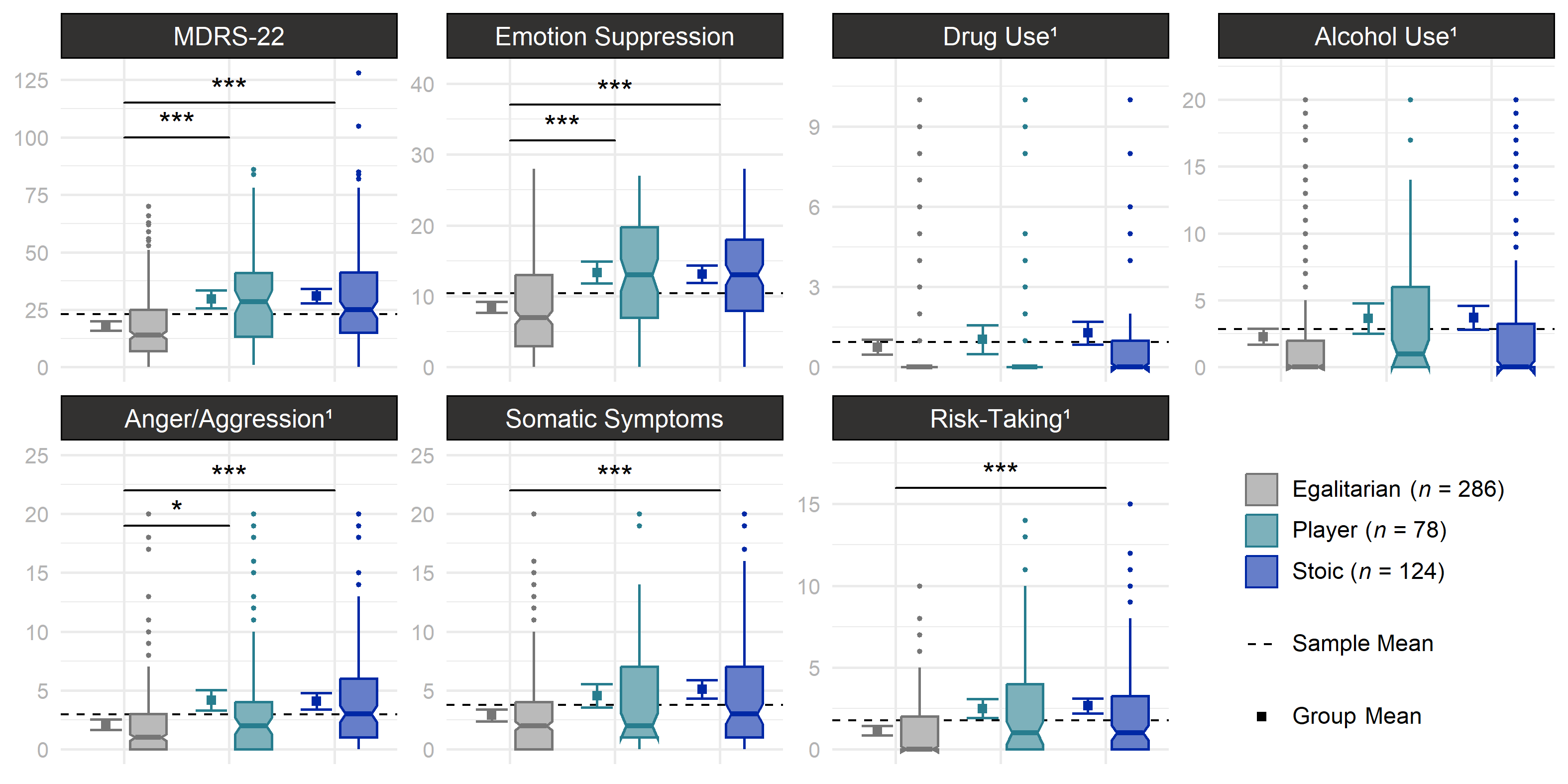 |
| *Note.* Group differences in externalizing depression symptoms assessed with the Male Depression Risk Scale (MDRS-22). *p*-values were adjusted for multiple testing using the Holm-method while 95% Wald confidence intervals (vertical error bars around the mean and notches around the median) are unadjusted. *n* = number of participants.  ^1^ Non-parametric Wilcoxon rank-sum test was used. ^*^ *p* < .05; ^***^ *p* < .001 |

| **Table S8**  *Odds Ratios of Hierarchic Regression Models 1–4 for Lifetime Suicide Attempt* | | | | | | | | | | | | |
| --- | --- | --- | --- | --- | --- | --- | --- | --- | --- | --- | --- | --- |
| **Predictor** | **Model 1** | | | **Model 2** | | | **Model 3** | | | **Model 4** | | |
|  | OR | 95% CI | | OR | 95% CI | | OR | 95% CI | | OR | 95% CI | |
| **TMI Profiles** |  |  | |  |  |  |  |  |  |  |  |  |
| Egalitarian (Intercept) | **0.10^***^** | [0.07, 0.16] | | **0.08^***^** | [0.05, 0.12] | | **0.14^***^** | [0.06, 0.30] | | **0.15^***^** | [0.07, 0.33] | |
| Player | 1.74 | [0.84, 3.62] | | 1.72 | [0.82, 3.61] | | 1.74 | [0.81, 3.72] | | 1.49 | [0.68, 3.25] | |
| Stoic | **2.54^**^** | [1.42, 4.58] | | **2.64^**^** | [1.45, 4.79] | | **3.10^**^** | [1.66, 5.81] | | **2.67^*^** | [1.40, 5.10] | |
| **Mental Health** |  |  |  |  |  |  |  |  |  |  |  |  |
| Depression Diagnosis |  |  |  | **2.54^*^** | [1.33, 4.84] | | 1.91 | [0.98, 3.71] | | 1.84 | [0.94, 3.58] | |
| Psychotherapy Use |  |  |  | 1.05 | [0.52, 2.12] | | 1.43 | [0.69, 2.96] | | 1.39 | [0.67, 2.89] | |
| **Sociodemographics** |  |  |  |  |  |  |  |  |  |  |  |  |
| Age^1^ *(years)* |  |  |  |  |  |  | 1.52 | [1.12, 2.05] | | **1.56^*^** | [1.15, 2.13] | |
| Income^1^ *(CHF)* |  |  |  |  |  |  | 0.59 | [0.34, 1.01] | | 0.59 | [0.34, 1.03] | |
| Tertiary Education |  |  |  |  |  |  | 0.60 | [0.33, 1.08] | | 0.58 | [0.31, 1.05] | |
| In a Relationship |  |  |  |  |  |  | 0.82 | [0.47, 1.44] | | 0.82 | [0.47, 1.45] | |
| Heterosexual |  |  |  |  |  |  | 0.57 | [0.30, 1.08] | | 0.56 | [0.30, 1.05] | |
| **MC-SDS** |  |  |  |  |  |  |  |  |  | 0.76 | [0.56, 1.02] | |
| **Fit and Performance** |  |  |  |  |  |  |  |  |  |  |  |  |
| χ^2^ (df) | 9.86 (2) | | | 10.98 (2) | | | 16.48 (5) | | | 3.49 (1) | | |
| Δ*R*^2^ *(in %)* | **3.68^**^** | | | **4.01^**^** | | | **5.85^**^** | | | 1.21 | | |
| AUC *(in %)* | 60.98 | | | 68.47 | | | 74.97 | | | 76.02 | | |
| Note. *p*-values were adjusted for multiple testing using the Holm-method while 95% Wald confidence intervals (95% CI) are unadjusted. OR = odds ratio; CI = Wald confidence interval; Δ*R*^2^ = difference in Nagelkerke's pseudo-*R*^2^; AUC = area under the curve; MC-SDS = MC-SDS = Marlowe–Crowne Social Desirability Scale. ^1^ variable was *z*-standardized. ^*^ *p* < .05; ^**^ *p* < .01; ^***^ *p* < .001 | | | | | | | | | | | | |
